# Conveying hypertension message: An investigation into the language and content used in primary health clinics in South Africa

**DOI:** 10.4102/phcfm.v12i1.2115

**Published:** 2020-02-13

**Authors:** Nokuthula G. Nkosi-Mafutha, Hester C. de Swardt, Sophie Mogotlane

**Affiliations:** 1 Department of Nursing Education, Faculty of Health Sciences, Tshwane University of Technology, Pretoria West, Pretoria, South Africa; 2 Health Studies, University of South Africa, Pretoria, South Africa

**Keywords:** hypertension, health education, language, health promoters, primary healthcare clinics

## Abstract

**Background:**

Hypertension is a global health burden affecting developed and developing countries, and South Africa is no exception.

**Aim:**

This article aims to highlight the language and content used in health education on hypertension in primary healthcare (PHC) by health promoters and in pamphlets.

**Methods:**

The study design was quantitative descriptive. The population comprised a purposive selected sample of 12 health promoters in 12 PHC clinics and 50 pamphlets relating to health education on hypertension. An audio recorder was used to record health education provided by health promoters. Quantitative content analysis and frequency distribution was used to analyse the data.

**Results:**

The health promoters used various South African languages mixed with English (code switching). Patients were taught about lifestyle modifications and encouraged to adhere to management therapy. The switching in language usage may affect the understanding of those who do not speak the local language and that may explain the reason for lack of hypertension-suited life modification required by health education.

**Conclusion:**

It is important that heath education on hypertension should be standardised so that the content of health education in clinic A is similar to that in clinic B. Information contained in pamphlets should be summarised and standardised to the content presented by health promoters.

## Introduction

Hypertension is primarily a condition of developing countries as two-thirds of the population suffering from hypertension lives in these countries.^1^ Hypertension is a ‘silent killer’ as many people do not realise that they have this condition because it often does not have warning signs or symptoms.^1^ In South Africa, more than six million people are suffering from hypertension.^2^ For some patients, the first contact with this condition was when they suffered a cerebrovascular accident.^3^ In addition, hypertension if not managed properly could lead to blindness, cardiac and kidney failure.^4^

Hypertension is a chronic non-communicable but preventable disease with approximately 17 million patient visits to South Africa’s primary healthcare (PHC) clinics for consultation of non-communicable diseases.^4^ Patients diagnosed with hypertension initially are encouraged to adhere to lifestyle modifications to enhance control and management of the condition as part of its non-pharmacological management. Thereafter, if hypertension remains uncontrolled, patient is required to use daily medication for the remaining period of their life.^4^ The National Department of Health (DoH) of South Africa recommends that health professionals practicing in PHC clinics provide health education to enhance compliance with the management and control of hypertension.^5^

Health education is a learning experience that fosters motivation, skills and self-efficacy necessary to improve health and facilitate behavioural changes.^5,6,7^ Health education uses communication processes such as individual discussions and mass and group media to reach target groups and includes printed health education materials, such as pamphlets and posters, to enhance learning.^7^ Unfortunately, low literacy levels, which are still a reality in the South African context could affect patient’s ability to comply with treatment. Reading and understanding the written messages used for pamphlets could be a challenge in a country such as South Africa that has 11 official languages, sign language and approximately 10 foreign languages used by immigrants from neighbouring African countries.^8^ In addition, low literacy could impair patient’s ability to read food labels and appointment dates written on clinic cards.^9^

For successful management and treatment of patients, the healthcare practitioner needs to avoid medical terminology and jargon and be clear and concise, and portray explicit factual content in a language comprehensible to the persons intended to receive the message.^10^ Patients need to know precisely what lifestyle modifications they need to focus on and understand why medication is needed and which medication is prescribed to actively participate in their own care. Unfortunately, providing health education does not necessarily mean comprehension and behavioural changes. For instance, Nkosi and Wright^11^ in a study conducted in Tshwane, which included 101 participants from three PHC clinics, found that patients acknowledged that they had received health education on hypertension; however, their blood pressure was not well controlled as their adherence to lifestyle modifications was poor.^11^ The literacy level of respondents could have played a major role in poor compliance, as their educational levels were lower than what was reported in the 2001 census, and this lower level of literacy could have led to poor understanding.^8^

According to the US Department of Health and Human Sciences^12^ there is a large discrepancy between the health information people receive and what they understand because of the complex way the information is presented and the use of unfamiliar terms and health-related jargon. Using plain language seems to be a promising strategy to overcome this barrier.^12^ In South Africa, health promoters are currently part of the PHC re-engineering serving under the stream ward-based PHC outreach teams led and managed by a professional nurse who provides guidance.^13^ Their main aim is to promote health and prevent ill-health through a variety of interventions. However, it is important to note that during data collection for this study, the process was not in place in those PHC clinics, therefore all the limitations that have been identified by Pillay and Baron^13^ concerning health promoters, such as the fact that these were not supervised, and were poorly trained with no clear mandate, were a concern.

The purpose of this study was to describe the language and the content used by health promoters during health education sessions in PHC clinics focusing on hypertension as well as the pamphlets provided to patients.

## Research design and methods

### Study design

The study followed a quantitative descriptive design. The content of health education on hypertension from pamphlets and as provided by health promoters during health education sessions at PHC clinics was described.^14^ The content was quantified by counting the number of times a word or phrase or concept was mentioned by health promoters and in pamphlets.^15^

### Study setting

The study was conducted in 12 PHC clinics situated in Tshwane municipality, Gauteng, South Africa. These clinics were responsible for the management of patients diagnosed with hypertension and treatment. Each of these clinics was allocated a health promoter whose main function was to provide health education to patients.

### Study population and sampling strategy

The population comprised health promoters in 12 PHC clinics and pamphlets or posters as unit of analysis relating to health education on hypertension. The non-probability purposive sampling method was used to select health promoters and pamphlets.^14^ This selection depended on the availability of health promoters and their willingness to participate, and on the availability of pamphlets or posters in each clinic during the period of data collection. The health promoter’s inclusion criteria stated, ability to provide consent to be audio-recorded as well as consenting to provide the researcher with two dates on which the researcher could record health education without notifying health promoter, this was exercised to prevent the Hawthorn effect.

## Validity and reliability of the study

The following measures were used to ensure reliability and validity:

- An audit trail was created to record data analysis decisions as they occurred.

For reliability, data were recorded on a normal working day, while health promoters were not expecting anyone to be recording them, this was done to prevent them from behaving in a certain manner. A trained field worker recorded the session, so they wouldn’t see the researcher on that specific day to prevent the health promoter acting in a certain manner. To ensure reliability, this was managed as health promoters agreed that they would be focusing on hypertension health education on two dates, and they allowed the researcher to visit the clinic and record health education session on any of the two dates.

- Field workers were trained prior to data collection to prevent the Hawthorn effect.
- An independent coder confirmed the data by checking it against audios and transcripts, and analysed the data to confirm emerging categories.

## Data collection

From the 12 PHCs, only 12 health promoters gave consent to participate, and data were collected through audio recording of the delivered health education session on hypertension. Pamphlets were collected each clinic immediately after health promoters completed their health education sessions. Fifty pamphlets related to health education on hypertension were collected, and after removing duplicates, only 11 pamphlets were left for the study.

## Data analysis

Data were analysed using quantitative content analysis and descriptive statistics for frequency distribution. Quantitative content analysis focuses on the presence of certain words, concepts, themes, phrases, characters or sentences in texts or sets of texts and to quantify this presence in an objective manner.^15^ Frequency distribution was used to calculate for how many times a word, phrase and concept appeared in the text. Table 1 describes the process followed to analyse the data using quantitative content analysis.

**TABLE 1:** Data collection process.

Recordings of health promoters	Pamphlets/posters
The 12 recorded hypertension health education sessions of health promoters were transcribed.	The 50 collected hypertension health education materials were assessed for duplication and only 11 printed materials (pamphlets and posters) remained for analysis.
For effective coding and data analysis, the recording that was transcribed first was numbered, meaning all the transcriptions were numbered 1–12.	The pamphlets and posters were numbered 1–11.
Themes for analysis were identified, guided by literature review regarding the subject of health education content on hypertension. The themes were as follows: definition of hypertension risk factors signs and symptoms pharmacological management non-pharmacological management complications of poorly controlled hypertension.	
Colour codes were used for each theme for effective coding.	
Transcript 1 was analysed by colour coding and categorising according to the colour of appropriate theme, e.g. red for prevention, blue for management and yellow for control.	
Formulating sub-categories: Transcript 1 served as point of departure for creating a frequency distribution list. For example, the first time a word, phrase or concept came up, such as ‘salt’, it would be highlighted blue, put under the theme ‘Non-pharmacological management’ and numbered as 1 as it would be the first time it was mentioned by a health promoter. If the same word, phrase or concept was mentioned by the second health promoter, it would be highlighted blue and numbered as 2. Should the same word, phrase or concept be mentioned by the fourth health promoter, it would be numbered as 3. See Addendum 1 for the list of words, phrases and concepts and frequency distribution resulting from the health education on hypertension provided by health promoters. For pamphlets and posters, the same principle was followed as for the recordings.	
The findings of oral health education and printed material on hypertension were combined and a word list according to syllables was created. This list of words, phrases and concepts was presented to the first panel of experts following the Modified Delphi Technique, selected the most common words, phrases and concepts used during health education on hypertension in order to develop a Hypertension Health Literacy Assessment Tools.^16^	

### Ethical considerations

The research was based on the three principles of Belmont Report concerning ethical conduct during research.^17^ The principles were beneficence, respect for human dignity and justice.^17^

Approval to conduct the study was obtained from the Research Ethics Committee of the University Faculty of Higher Degrees Committee (#FREC2013/06/001 (2) (SCI)). Written permission to conduct the study was obtained from the Gauteng Department of Health (DoH); the managers of PHC clinics also gave their permission for the research to be conducted. The health promoters were provided with information leaflet, and written informed consent was obtained before commencement of recording of their health education sessions.

## Results

The results are presented in terms of the demographics of health promoters, the languages used and the content of the hypertension-related health education sessions (i.e. what is being taught to patients by healthcare promoters).

### Characteristics of health promoters and languages used during hypertension-related health education sessions

All health promoters (*n* = 12) had passed Grade 12 and were employed for more than five years. All collected pamphlets (*n* = 11) were in English. The languages used by health promoters during health education mixed with English were Setswana (seven promoters), Sepedi (two promoters) and IsiNdebele (three promoters).

### Content of hypertension-related health education

Table 2 contains the content in terms of words/phrases/concepts that were commonly used during health education on hypertension by health promoters and pamphlets. These were categorised in terms of definition of blood pressure and hypertension, risk factors, signs and symptoms, pharmacological and non-pharmacological management, and complications of uncontrolled hypertension.

**TABLE 2:** List of commonly used words or phrases or concepts during hypertension health education by health promoters and from pamphlets and posters.

Theme	Categories	Subcategories	Frequency			
			Health promoters		Pamphlets	
			(*n* = 12)	%	(*n* = 11)	%
Blood pressure	Definition of blood pressure	Blood pressure	-	-	-	-
	Definition of hypertension	Blood pressure	6	50	4	33
		Silent killer	3	25	2	18
	Risk factors for developing or aggravating hypertension	Stress	7	58	3	27
		Genetic predisposition	5	42	1	9
		Lot of salt and spice	5	42	2	18
		Overweight	3	25	5	45
		Oily food	5	42	0	0
	Signs and symptoms of hypertension	Headaches	5	42	2	18
		Dizziness	5	42	2	18
		Tiredness and weakness	3	25	0	0
	Pharmacological management of hypertension	Taking medications correctly (comply with prescribed treatment)	9	75	6	55
		Do not borrow each other’s medication	2	17	1	9
		Take treatment even when feeling better	0	0	3	27
	Non-pharmacological management of hypertension	Aromat^®^ and spices, all have salt, do not eat	7	58	0	0
		Do not eat salt	4	33	0	0
		Reduce salt, use salt sparingly	4	33	4	33
		Reduce oil	4	33	3	27
		Eat fruits	4	33	4	33
		Eat vegetables	9	75	4	33
		Drink water	7	58	1	9
		Exercise for 30 min (regular)	3	25	6	55
		Running, playing tennis, walking fast, cooking	10	83	0	0
		Reduce stress levels	8	67	0	0
		Join support groups	3	25	0	0
		Eat healthy (spinach, peanuts, herbs, less starch, proteins)	6	50	0	0
		Stop smoking cigarettes	4	33	8	73
		Stop alcohol	1	8	0	0
		Moderate alcohol	0	0	7	64
		Keep appointments	0	0	2	18
	Complications of uncontrolled hypertension	Stroke	8	66	6	55
		Death	3	25	2	18
		Poor eye sight (blindness)	2	17	2	18
		Kidney problems/failure	0	0	4	33
		Heart attacks	1	8	4	33

## Discussion

### Language used in pamphlets and during health education sessions

All (*n* = 11; 100%) printed health education materials found in the PHC clinics were written in English. This could be a limitation for patients who are not able to read or speak English and have no one to read for them at home; moreover, the pamphlet remains the only point of reference from the clinic. The health promoters used native languages mixed with English in their health education sessions. Even though it is not entirely ineffective to use native languages during health education, the implication of this is that patients might not adequately understand what is being taught, which consequently affects their daily self-management.^11^ In another study performed in Gauteng PHC, the researchers discovered that although patients were provided health education, the study’s results revealed noncompliance with hypertension management and control, specifically poor compliance of dietary practices and poor knowledge of food containing high salt and fat.^11^

Another challenge with language use was that immigrants who might not understand any of the 11 languages and even the sign language could miss valuable information provided by health promoters when they switched to native language, which was mostly Setswana as the commonly spoken language in Tshwane. In addition, health promoters might not speak all 12 official languages, meaning that some patients who spoke other South African language might miss important information because of language barrier. Thus, they have to rely on the already limited summarised information in pamphlets if they know English, otherwise they would not be able to access complete information.

### Content of hypertension-related health education from health promoters and pamphlets

For the theme ‘Definition of Hypertension’, the most common term used for hypertension was blood pressure, as explained by 50% (*n* = 6) of health promoters. However, blood pressure is not an accurate translation of hypertension.

For the theme ‘Non-pharmacological Management of Hypertension’, overweight was least mentioned; it was used by only 25% (*n* = 3) of health promoters and in 41.6% (*n* = 5) of pamphlets on hypertension. Obesity or overweight, especially adipose tissue, is a major contributing factor to the development and complications of hypertension and type 2 diabetes.^4^ In a study conducted in the Thulamela area of Limpopo province in South Africa, people viewed overweight as a sign of being healthy and taking proper care of health.^18^ This wrong notion indicates how patients view their weight in relation to hypertension as an illness and might not even be convinced for weight loss. This means that the person providing health education must understand the target population, including their cultural background, as this informs patients’ perception about their health.^19^

Furthermore 58% (n+7) of the health promoters’ health education content emphasised that salt and spices are risk factors that aggravate hypertension and should not be used at all. However, all pamphlets lacked this crucial information. Moreover, every health professional should know that food without spices and salt is tasteless, and patients are not easily motivated to stop using these.^20^ However, health promoter’s ability to clearly describe how salt and spices affect patient’s body and blood pressure would allow patient to internalise and to have a clear picture of the risk if they continue using salt and spices. The same could be carried out by health promoters when providing health education about quitting smoking. It is crucial to note that the health education content was also not clear about salt intake as 33.3% (*n* = 4) of health promoters and pamphlets said ‘use salt sparingly or reduce salt intake’, whilst 58% (*n* = 7) of health promoters advised not to use salt/spice at all; these two statements could confuse patients. A person should not eat more than half a teaspoon of salt a day regardless of what the person eats throughout the day.^21^ Health promoters and pamphlets were supposed to explain that increase in salt intake disrupts normal physiological mechanism required for sodium homeostasis, thereby causing increase in blood pressure. Similarly, increased renal re-absorption of sodium necessitates increased water re-absorption to maintain plasma sodium concentration at 140 mmol/L. Similarly, increased renal re-absorption of socium necessitates increased water re-absorption to maintain plasma socium concentration at 140 mmol/ L; this leads to an increased intravascular volume and increased cardiac output, should be sustained, hypertension develops.^22^

Although health promoters taught hypertensive patients to eat more fruits and vegetables, but failed to mention that fruits and vegetables should be eaten daily. Another risk factor for development of hypertension is tobacco sniffing and smoking. Tobacco, whether it is cigarette smoking or sniffing, is a risk factor for increased hypertension.^23^ Ceasing to smoke or eat tobacco could decrease at least 2 mmHg of blood pressure in people with hypertension.^24^ In this study, only 33.3% (*n* = 4) of health promoters mentioned to stop smoking as a non-pharmacological management of hypertension.

Health promoters need to educate patients with hypertension about smoking in relation to hypertension and not just in a general way. Similarly, drinking alcohol has to be discouraged in patients with hypertension. This was mentioned by only one (8.3%) health promoter whilst educating about hypertension and nothing was mentioned on this (0%) in pamphlets. Alcohol drinking should be limited to just one standard drink per day for women and two drinks per day for men.^25^ Although link between alcohol and blood pressure is unclear, it might be because of the stimulation of sympathetic nervous system and the inhibition of vascular relaxing substance.^26,27^

Exercise, including brisk walking, running, cooking and playing tennis, was mentioned by 83.3% (*n* = 10) of health promoters, but not in pamphlets, which are intended for reference at home. Reducing stress by joining support groups was mentioned as non-pharmacological management of hypertension, which is supported by scientific literature as well.^28,29^

In this study, headache 41.6% (*n* = 5), dizziness 41.6% (*n* = 5) and weakness and tiredness 25% (*n* = 3) were mentioned by health promoters as signs and symptoms of hypertension. However, most people initially do not experience these symptoms, but by the time person experiences headache, dizziness and weakness in any part of the body, hypertension has advanced to a chronic state for not being diagnosed or because it is poorly controlled.^28^ In South Africa, about 20% of men and women are hypertensive but the level of awareness about it is lower in men than in women, and if they are aware then the level of control is poor; this could be attributed to noncompliance of its management.^4^

Under the theme ‘Pharmacological Management’, compliance with medication was mentioned. Hypertension is one of those conditions where people might not see the need to comply to the medication and stop taking it, especially because they do not feel sick or have any signs of being sick.^29^ In this study, health promoters and pamphlets mentioned that patients should take their medication as prescribed but failed to add that medication should be continued on regular basis even when they do not feel sick.

To add to the treatment regimen, only 17% health promoters (*n* = 2) and 9% pamphlets (*n* = 1) mentioned that borrowing hypertension medication from friends or other people was an unacceptable practice. Literature reveals that patients with chronic conditions often share their medications.^30^ There could be quite a number of reasons that a patient has finished medication and is not in a position to get fresh stock, for example having to travel long distance to reach clinic, or medication not available even in clinic, or lack of money to purchase medicines if the public health facility failed to supply the same.^31^ It is important to note that the South African National Department of Health has an agreement with service providers such as Clicks Pharmacy to provide patients medicines nationwide for chronic illnesses.^32^ However, health promoters and pamphlets should observe that it is not acceptable for individuals to share their medications with other people and this should be discouraged because a person could have complications or poisoning by taking wrong medication or there could be drug–drug interaction because some medicines are not allowed to be taken with some specific medicines.^33^

Under the theme ‘Uncontrolled Hypertension’, health promoters and pamphlets noted strokes, blindness and death as the complications of uncontrolled hypertension. However, kidney failure, another complication of uncontrolled hypertension, was not mentioned by health promoters, but only in 33.3% (*n* = 4) of pamphlets. In South Africa, about 60%–65% adult population is suffering from kidney failure because of uncontrolled hypertension with a waiting period for kidney transplant being 6–12 years.^34^

### Implications of the findings and recommendations

Patients who are provided health education in PHC clinics might be missing much of information on account of language barriers and illiteracy. The government (DoH) should invest in pamphlets and posters in PHC clinics that are written in languages commonly used by the community. The presentation of these pamphlets should not be academic but professional with decent illustrations. Organisations should conduct seminars and workshops for health promoters on Continuous Professional Development (CPD) programmes to keep abreast of developments in hypertension-related health education. The selection criteria to become a health promoter need to be revisited; this is similar to the findings of Peu and Mthombeni^35^ who in their study investigated the needs of health promoters. Health promoters should advocate for printed hypertension-related health education material that are easy to read (preferably of grade 4 reading level) and culturally relevant for patients. A lesson plan of health education on hypertension should be designed by health promoters and be reviewed on monthly basis.

## Limitations

The limitation of the study could be that the health promoters who participated in the study might have had limited knowledge about hypertension. The health promoters gave two possible dates for when they would be providing health education; therefore, the authors tried to prevent bias by obtaining two dates, to use one of the dates to record the health education. On these two dates, the health promoters might have changed their behaviour, leading to desirability bias.

## Conclusion

Language in health education is an important factor. Even though health promoters did the right thing by providing health education in the language most spoken in each PHC clinic, immigrants using clinics must be provided with the translation so that they could also benefit from health education. Health information in pamphlets should be available in all possible local languages so that patients can refer to pamphlets in case they forget what was taught in the clinic. The results from the content taught by health promoters and the matter in pamphlets reflect that information provided about hypertension could differ, which could be confusing. For example, if a patient was told to reduce salt intake in clinic A and next week on visiting clinic B was told to stop taking salt, these two statements could be confusing for the patient, and he might even lose trust in health promoters as well as pamphlets.

Pamphlets did not contain all the relevant information provided by health promoters. Therefore, it is important that heath education on hypertension should be standardised so that the content of health education taught in all clinics, including in clinics A and B, should be the same. Information contained in pamphlets should be summarised and standardised with the content presented by health promoters. Additionally, pamphlets should be approved by DoH and there must be a guideline regarding the content of pamphlets. The South African DoH needs to control and regulate the information provided to patients, and to make sure that furnished information is up to date, precise and fits South Africans and their lifestyles.
